# Precise Aperture-Dependent Motion Compensation with Frequency Domain Fast Back-Projection Algorithm

**DOI:** 10.3390/s17112454

**Published:** 2017-10-26

**Authors:** Man Zhang, Guanyong Wang, Lei Zhang

**Affiliations:** 1 School of Software, Xidian University, Xi’an 710071, China; mzhang@xidian.edu.cn; 2 National Laboratory of Radar Signal Processing, Collaborative Innovation Center of Information Sensing and Understanding, Xidian University, Xi’an 710071, China; 3 Beijing Institute of Radio Measurement, The Second Academy of China Aerospace Science and Industry Corporation (CASIC), Beijing 100854, China

**Keywords:** synthetic aperture radar (SAR), motion compensation (MOCO), frequency domain fast back-projection algorithm (FDFBPA), chirp-z transform (CZT)

## Abstract

Precise azimuth-variant motion compensation (MOCO) is an essential and difficult task for high-resolution synthetic aperture radar (SAR) imagery. In conventional post-filtering approaches, residual azimuth-variant motion errors are generally compensated through a set of spatial post-filters, where the coarse-focused image is segmented into overlapped blocks concerning the azimuth-dependent residual errors. However, image domain post-filtering approaches, such as precise topography- and aperture-dependent motion compensation algorithm (PTA), have difficulty of robustness in declining, when strong motion errors are involved in the coarse-focused image. In this case, in order to capture the complete motion blurring function within each image block, both the block size and the overlapped part need necessary extension leading to degeneration of efficiency and robustness inevitably. Herein, a frequency domain fast back-projection algorithm (FDFBPA) is introduced to deal with strong azimuth-variant motion errors. FDFBPA disposes of the azimuth-variant motion errors based on a precise azimuth spectrum expression in the azimuth wavenumber domain. First, a wavenumber domain sub-aperture processing strategy is introduced to accelerate computation. After that, the azimuth wavenumber spectrum is partitioned into a set of wavenumber blocks, and each block is formed into a sub-aperture coarse resolution image via the back-projection integral. Then, the sub-aperture images are straightforwardly fused together in azimuth wavenumber domain to obtain a full resolution image. Moreover, chirp-Z transform (CZT) is also introduced to implement the sub-aperture back-projection integral, increasing the efficiency of the algorithm. By disusing the image domain post-filtering strategy, robustness of the proposed algorithm is improved. Both simulation and real-measured data experiments demonstrate the effectiveness and superiority of the proposal.

## 1. Introduction

The atmospheric turbulence disturbs the ideal trajectory of aircraft during the whole process of flight, and this causes not only serious blurring, but also geometric distortion of the synthetic aperture radar (SAR) [1,2,3] imagery. Therefore, motion compensation (MOCO) [4,5,6] is an essential processing procedure for airborne SAR imaging. For the efficiency and accuracy of MOCO, a high-precision inertial navigation system (INS) is commonly mounted on the platform to record the real-time velocity and position information. Therefore, the MOCO accuracy prominently relies on the MOCO strategies., Effectively compensating the residual motion error is still a problem worth studying, especially for millimeter-wave band SAR imagery.

As analyzed in [7], it is clear that the main difficulty of MOCO is sourced from the space-variance of the motion error. The conventional two-step MOCO method [6] is proposed to compensate the range-variant motion error, which is widely applied by embedding MOCO into the SAR algorithms [8]. However, the two-step MOCO method neglects the residual azimuth-variant motion errors, thus the focusing performance decreases for SAR imagery with wide beam and high resolution, especially when the atmospheric turbulence is severe. In order to solve this problem, several effective algorithms [9,10,11,12,13,14,15] have been developed in the current literature, which compensate for the residual azimuth-variant motion error in different ways. The sub-aperture topography- and aperture-dependent algorithm (SATA) [10,11] and precise topography- and aperture-dependent motion compensation algorithm (PTA) [13], are typical examples. Sliding sub-aperture processing is an essential tool in both of the azimuth-variant MOCO strategies, which calculates the residual azimuth-variant motion error relative to the center of each sub-aperture. A set of spatial filters are then established to remove the residual motion errors. In SATA, the time-varying residual error within an azimuth-time sub-aperture is approximated as a constant, and the residual motion errors are corrected in the Doppler domain under a Doppler-to-angle map. On the other hand, PTA corrects the residual spatial-variant motion errors in the image domain at a price of some efficiency losses. In PTA, the coarse-focused image is divided into overlapping sub-blocks, and the residual motion error relative to every block center is compensated for by a post-filtering strategy. Because of its high accuracy, PTA is one of the most widely used algorithms for azimuth-variant MOCO in real airborne SAR imagery. In general, PTA performs well in normal conditions, such as that when the motion errors are not very severe. However, a significant problem arises in the case of strong motion errors, such as SAR imaging under unmanned aerial vehicle (UAV) [16,17] platforms or serious atmospheric situations. In these cases, PTA would evidently expose its shortcoming. Image domain post-filtering of PTA needs to segment the image into sub-blocks. Because this strategy compensates the azimuth-variant motion error block-to-block, it confronts three main problems when dealing with sever motion errors. Firstly, energy diffusion of point targets is more severe in image domain, therefore, the sub-block size needs to be extended to make sure that the whole diffused energy of each effective target point is included in either of the adjacent sub-blocks. Otherwise, the target cannot be fully refocused with partial defocused energy in each sub-block, together with ghost shadows after block stitching. Secondly, image domain post-filtering assumes that azimuth-variant motion errors in one azimuth block are the same as the block center point; this hypothesis is not consistent with strong motion errors. Therefore, the robustness of PTA would evidently decrease. A solution is to extend the overlapping part between adjacent sub-blocks. Thirdly, with the significant extension of sub-block size and overlapping ratio between the neighboring sub-blocks for PTA post-filtering, the post-filtering strategy of PTA faces an increasing calculation burden, which is approximately as complex as conventional the back-projection algorithm. Inevitably, PTA has to make a balance between image quality reduction and increased calculation. The motivation behind the current study is the desperate need for an imaging method with both high precision and high efficiency for practical applications with strong motion errors.

Aiming to solve the PTA problems mentioned above, a frequency domain fast back-projection algorithm (FDFBPA) is proposed in this paper. Instead of post-filtering for a sub-block in the image domain, FDFBPA compensates the residual azimuth-variant motion errors by precisely calculating the azimuth matched filtering (AMF) [15] function, and using the fast back-projection process in the azimuth wavenumber domain. FDFBPA could be thought of as an extension of previous work [18,19], to achieve an effective spatial-variant MOCO. In FDFBPA, a precise AMF function with motion errors is derived. The spectrum is uniformly partitioned in the azimuth wavenumber domain and each sub-aperture is back-projected to obtain a set of coarse resolution images. Then, sub-images are fused in the azimuth wavenumber domain in order to achieve a full resolution image. Moreover, by introducing a linear Doppler approximation in the AMF, sub-aperture back-projection integral is implemented by the fast chirp-Z transform (CZT) [20,21], yielding promising efficiency enhancement for the algorithm. Compared with PTA, FDFBPA promises fully focused images with high efficiency and robustness, and is suitable for real airborne SAR imagery.

We organized this paper as follows: Section 2 gives the geometry model and calculates the precise expression of signal with residual azimuth-variant motion error in azimuth wavenumber domain, and illustrates the shortcoming of post-filtering algorithms; in Section 3, the principle of FDFBPA is illustrated in detail; Section 4 shows a flowchart of the proposed FDFBPA and analyzes computation burden; in Section 5, extensive experimental results with both simulated and real Ka-band airborne SAR data are given; conclusions are drawn in the last section.

## 2. Signal Model and Conventional Post-Filtering Algorithms

**Figure 1:**
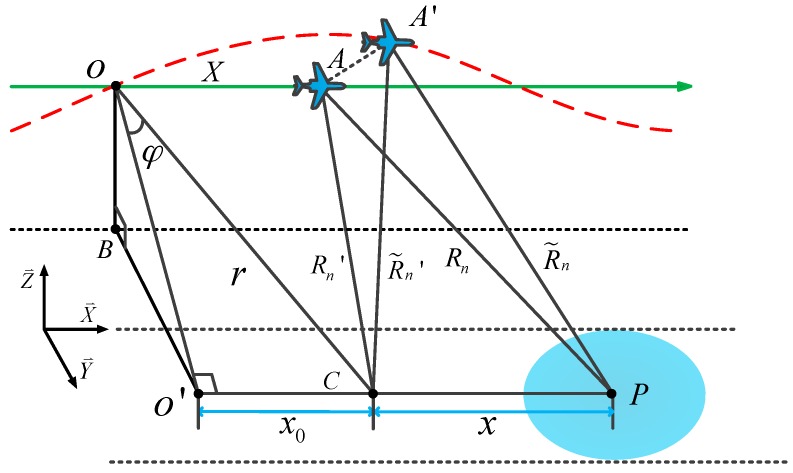
Real SAR imaging geometry.

Real SAR imaging geometry is given in Figure 1. The geometry is defined in a rectangular system as $O-\vec{X}\vec{Y}\vec{Z}$, where O is the origin of coordinates and $\vec{X}$, $\vec{Y}$ and $\vec{Z}$ indicate the long track direction, cross track direction and height direction, respectively. In an ideal case, radar platform travels along a straight line with a constant velocity V, shown as the solid line along the track. Because of atmospheric turbulence, real trajectory is a curve shown as the dotted line across the track. During the data acquisition process, a radar beam illuminates the ground with a squint angle φ. Point C denotes the scene center with the long track coordinate as $x_{0}$, and the distance between O and C is indicated as r. Symbol P stands for the target located on the scene center line O′C, which is parallel to the trajectory. Distance between target P and scene center C is given by x. The echo expression from P is given by [22]:

(1) $$s\left(\tau ,X\right)=\varepsilon_{p}\cdot \mathrm{rect}\left(\frac{\tau -\Delta t}{T_{p}}\right)\cdot \mathrm{rect}\left(\frac{X-x_{0}-x}{L}\right)\cdot \exp\left[j2\pi \left(-f_{c}\Delta t+\frac{\alpha {\left(\tau -\Delta t\right)}^{2}}{2}\right)\right]$$

where, $\varepsilon_{p}$ corresponds to the complex valued scattering amplitude of the point target, τ denotes the range fast-time, $T_{p}$ denotes the pulse duration width, L denotes the synthetic aperture length, $f_{c}$ is the center frequency, α is the signal chirp rate, $\Delta t=2R_{n}/c$ stands for the round-way propagation time between target P and radar and c denotes the speed of light. Symbol rect(⋅) denotes the rectangular window function and X represents the along track position of flight. Ignoring the effects of motion error, the slant range $R_{n}$ is expressed as:

(2) $$R_{n}\left(X,x,r\right)=\sqrt{{\left(r\cos\varphi \right)}^{2}+{\left(X-x_{0}-x\right)}^{2}}$$

Actually, the real slant range deviates from the ideal $R_{n}$ for non-ignorable motion error, which is shown as ${\tilde{R}}_{n}$ in Figure 1. The deviation blurred slant range ${\tilde{R}}_{n}$ contains four main components: ideal $R_{n}$, range- and azimuth-invariant motion error Δr, range-variant motion error $\Delta r_{b}$ and azimuth-variant motion error $\Delta r_{\varepsilon }$. In order to compensate for motion errors during the imaging process, a two-step MOCO [6] method is widely used. It achieves range-dependent MOCO for raw data, where the first step is bulk MOCO to compensate, and the second step is range-variant MOCO to compensate. However, the remaining azimuth-variant motion error is a considerable factor, especially for SAR systems with a wide aperture in azimuth. It is not difficult to understand azimuth-variant motion error. In Figure 1, it is shown that $R_{n}$ and ${\tilde{R}}_{n}$ are ideal and real slant range from aircraft to point P, while $R_{n}\prime$ and ${\tilde{R}}_{n}\prime$ are ideal and real slant range from aircraft to scene center C. The two-step MOCO method compensates for the whole scene with motion error of $\Delta R\prime ={\tilde{R}}_{n}\prime -R_{n}\prime$. However, with respect to point P, the actual motion error is $\Delta R={\tilde{R}}_{n}-R_{n}$. It is obvious that ΔR≠ΔR′ because of the different projection directions of aircraft deviation, which is the cause of azimuth-variant motion errors. We can calculate the azimuth-variant motion error $\Delta r_{\varepsilon }$ by $\Delta r_{\varepsilon }=\Delta R\prime -\Delta R$. 

Ignoring the envelope terms of Equation (1), after processing by range cell migration correction (RCMC) [3] and two-step MOCO, the expression of range compressed signal is expressed by [14]:

(3) $$s_{t}\left(X,x,r\right)=\exp\left\{-jK_{rc}\left[R_{n}\left(X,x,r\right)+\Delta r_{\varepsilon }\left(X,x,r\right)\right]\right\}$$

where $K_{rc}=4\pi /\lambda$, λ denotes the wavelength and $\Delta r_{\varepsilon }$ is the residual azimuth-variant motion error. We omitted the detailed derivation procedures of RCMC, two-step MOCO and range compression from (1) to (3), which are illustrated in [3]. The azimuth wavenumber domain signal would be obtained through an azimuth Fourier transform to (3), which is given by:

(4) $$\begin{array}{ll} S_{f}(K_{x},x,r) & =\int s_{t}\left(X,x,r\right)\cdot \exp\left(-jK_{x}X\right)dX\\ & \;=\int \exp\left\{-jK_{rc}\left[R_{n}\left(X,x,r\right)+\Delta r_{\varepsilon }\left(X,x,r\right)\right]-jK_{x}X\right\}dX \end{array}$$

where $K_{x}$ denotes the azimuth wavenumber spectrum, with $-K_{a}/2\leq K_{x}\leq K_{a}/2$ and $K_{a}$ denotes the spectrum range.

For simplification of the derivation, the principle of the stationary phase (POSP) [3] is used to approximately solve the integration operation in Equation (4). During calculation of the stationary phase point in (4), it is noted that because the ideal relationship between Doppler frequency and instantaneous radar slight angle is corrupted by the residual azimuth-variant motion errors, the azimuth wavenumber spectrum is also distorted. In order to acquire the precise time–frequency relationship, residual azimuth-variant motion errors need to be taken into consideration. Therefore, precise stationary phase point $X^{*}$ would be solved by the equation as follows [15]:

(5) $$\frac{\partial \left(R_{n}+\Delta r_{\varepsilon }\right)}{\partial X}+\frac{K_{x}}{K_{rc}}=0$$

For the propose of deducing an explicit expression of $X^{*}$, $R_{n}$ in (2) is expanded into fourth order polynomial shown as follows:

(6) $$R_{n}\left(X,x,r\right)\approx r-\frac{x_{0}}{r}\left(X-x\right)+\frac{\cos^{2}\varphi }{2r}{\left(X-x\right)}^{2}+\frac{\cos^{2}\varphi \sin\varphi }{2r^{2}}{\left(X-x\right)}^{3}-\frac{\cos^{2}\varphi \left(1-4\sin^{2}\varphi \right)}{8r^{3}}{\left(X-x\right)}^{4}$$

Due to the fact that low-order components usually dominate the residual motion error function, the azimuth-variant motion error $\Delta r_{\varepsilon }$ is expressed by the Taylor expansion as follows:

(7) $$\Delta r_{\varepsilon }\left(X,x\right)\approx a_{0}+a_{1}\left(X-x\right)+a_{2}{\left(X-x\right)}^{2}+a_{3}{\left(X-x\right)}^{3}+a_{4}{\left(X-x\right)}^{4}$$

where $a_{0}\cdots a_{4}$ represent the polynomial fitting parameters. It is important to mention that $\Delta r_{\varepsilon }$ is related to range r, and that $a_{0}\cdots a_{4}$ are also range dependent. We omit range variable r here in order to simplify the expression. According to previous research in [15], the method of series reversion (MSR) [23] can be utilized to obtain a precise expression of the stationary phase point $X^{*}$, which is given by:

(8) $$X^{*}=p_{1}y+p_{2}y^{2}+p_{3}y^{3}+x$$

where

(9) $$p_{1}=\frac{\cos^{2}\varphi }{r}+2a_{2}$$

(10) $$p_{2}=\frac{3\cos^{2}\varphi \sin\varphi }{2r^{2}}+3a_{3}$$

(11) $$p_{3}=-\frac{\cos^{2}\varphi \left(1-4\sin^{2}\varphi \right)}{2r^{3}}+4a_{4}$$

(12) $$y=-\frac{K_{x}}{K_{rc}}-a_{1}+\frac{x_{0}}{r}$$

The stationary phase point $X^{*}$ takes azimuth-variant motion errors into consideration, and thus, a precise expression in the azimuth wavenumber domain is obtained as follows:

(13) $$S_{f}\left(K_{x},x,r\right)=\exp\left\{-jK_{rc}\left[R_{n}\left(X^{*},x,r\right)+\Delta r_{\varepsilon }\left(X^{*}\right)\right]-jK_{x}X^{*}\right\}$$

**Figure 2:**
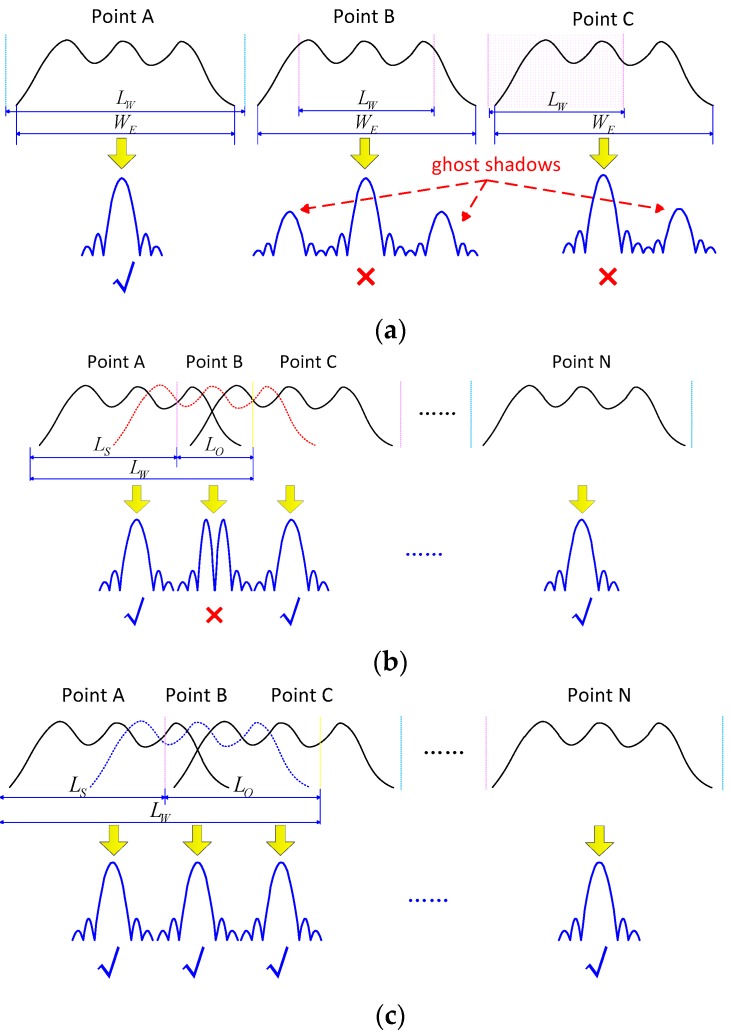
Illustration of sliding parameters for the post-filtering strategy: (**a**) Window length restriction for single point; (**b**) A failure case of sliding parameters for point array; (**c**) A successful case of sliding parameters for point array.

According to the analyses shown above, one can note that the azimuth-shift invariance of azimuth wavenumber spectrum is destroyed in the presence of the residual azimuth-variant phase error. The conventional azimuth-matched filtering function would fail to focus the image completely in this case. As developed in [13], PTA, which compensates for the residual azimuth-variant phase error using a post-filtering strategy, is an effective approach to deal with the problem. Its main focusing flow is commonly divided into two stages, one is coarse focusing stage processed by conventional azimuth matched filtering, while the other is image refocusing stage achieved by sliding window compensation. However, in order to ensure the effectiveness of the post-filtering strategy, it needs to make a careful selection of the window length and overlapping range. In order to illustrate these constraints, explanatory drawings are shown in Figure 2. Figure 2a gives the refocusing condition of window length with respect to a single point. Points A, B and C are three defocusing points for the residual motion errors, the energy diverging width is denoted by $W_{E}$, and $L_{W}$ denotes the window length. It is clearly shown that Point A is able to be fully refocused because its spreading energy with blur is entirely contained within the block. However, it is not effective for points B and C which have truncated parts within neighboring blocks. Besides residual blur, the truncation would also emerge as ghosts in the image. Based on this analysis, we give the sub-image length restrain for PTA as follows:

(14) $$L_{W}\geq W_{E}$$

For the propose of analyzing the relationship between window length and the overlapping range, Figure 2b also illustrates a case of the failure of sliding parameters for point array, $L_{S}$ denotes the sliding range and $L_{O}=L_{W}-L_{S}$ denotes the overlapped length. In this case, the overlapped length between the adjacent blocks is not long enough, so that points A and C both satisfy the refocusing condition, while point B, which is not fully contained in neither of the adjacent windows, and the refocused result of point B would be broken. In order to address this issue, the size of a block should be extended accordingly, which gives a successful case of sliding parameters for the point array in Figure 2c. In Figure 2c, the overlapped range between the adjacent blocks increases, indicating that point B could be focused successfully. In general, the relationship between block size and overlapping length is regulated as follows:

(15) $$L_{S}\leq L_{W}-W_{E}$$

It is important to mention that Equations (14) and (15) represent the relation of block size and overlapping parameters in a limited condition. A significant problem arises in the case of strong motion errors, such as SAR imaging under UAV platforms or serious atmospheric situations. The energy of a scatter in azimuth direction would be seriously diverged. In these cases, PTA would evidently expose its shortcoming, so the size of sub-block in PTA post-filtering should be extended dramatically, while the overlapping range between the neighboring blocks needs to be raised in order to satisfy the refocusing condition, which causes serious computational loads. Therefore, PTA has to make a balance between imaging quality reduction and inevitable increase in calculations. The motivation behind the current study is the desperate need for an imaging method with both high precision and high efficiency for practical applications with strong motion errors.

## 3. Frequency Domain Fast Back-Projection Algorithm (FDFBPA)

As is illustrated in Section 2, PTA removes the residual azimuth-variant motion errors by the sliding window post-filtering strategy. However, the data segmentation in the image domain seems not to be a wise choice when the residual motion error is severe. Because image domain blocking has to face the dilemma of balancing imaging quality and efficiency, this strategy is inapplicable for practical situations. In this section, we aim at providing a method for solving this problem in theory.

A. Precise Frequency Domain Back-Projection Algorithm (FDBPA)

In order to ensure compensation precision, a point-to-point strategy is preferred rather than the post-filtering block-to-block compensation method. FDBPA is more robust in dealing with strong motion errors, and is thought of as a precise point-to-point imaging method. This method is based on the precise wavenumber spectrum expression deduced in Equation (14), while each point in the imaging grid would be well-focused by adapting a precise back-projection integral. We briefly introduced the principle of FDBPA at the beginning of this section. Before the imaging process, a full resolution imaging grid is used for the FDBPA imaging process. We pick out one point P with coordinate $\left(x_{p},r\right)$, and to P, the echo in azimuth wavenumber spectrum is expressed as $S_{f}\left(K_{x},x_{p},r\right)$ which is shown in (13); so the precise AMF function phase $\Phi_{m}\left(K_{x},x_{p},r\right)$ is calculated by:

(16) $$\Phi_{m}\left(K_{x},x_{p},r\right)=K_{rc}\left[R_{n}\left(X^{*},x_{p},r\right)+\Delta r_{\varepsilon }\left(X^{*}\right)\right]+K_{x}X^{*}$$

Coherent accumulation of point P is realized by the back-projection integration in the wavenumber domain as follows:

(17) $$S\left(x_{p},r\right)=\int_{\Delta K_{x}/2}^{\Delta K_{x}/2}S_{f}\left(K_{x},x_{p},r\right)\cdot \exp\left[j\Phi_{m}\left(K_{x},x_{p},r\right)\right]dK_{x}$$

where, $\Delta K_{x}$ denotes the azimuth wavenumber spectrum width. It needs to calculate the corresponding AMF for a point-to-point back-projection accumulation for the other points. This FDFBPA process avoids image domain post-filtering, and provides high robustness even with serious motion errors. FDBPA achieves coherent accumulation in a point-by-point manner, and thus, its efficiency is low. In terms of this issue, several studies [24,25] have discussed how to increase the computational efficiency of the back-projection integral without focal quality loss.

B. Acceleration to FDBPA Process

According to our previous work in [18], the back-projection integral can be accelerated by the sub-aperture coarse imaging and coherent spectrum combination, and we find that the sub-aperture processing strategy is also applicable for acceleration. In this subsection, we investigate a further acceleration to the precise FDBPA process, and it is named FDFBPA. Below , we introduce the theory of acceleration method.

After RCMC processing, two-step MOCO and range compression to the raw data, the signal expression is shown in Equation (3), and after azimuth Fourier transform, the signal is then transformed to azimuth wavenumber domain shown in Equation (4). The procedure above is similar to FDBPA. The difference is that we can then uniformly partition the signal in the azimuth wavenumber domain based on the theory of sub-aperture strategy. In each sub-aperture procedure, a uniform coarse resolution imaging grid is constructed, which has the coordinate $\left(x_{sub},r\right)$ with $\;-x_{a}/2\leq x_{sub}\leq \;x_{a}/2$, where $x_{a}$ denotes the scene range in azimuth. Assuming the total sub-aperture number is U, as for the *u*th sub-aperture, the azimuth wavenumber spectrum center is $K_{xu}$, and wavenumber spectrum length is $\Delta K_{xa}$. For a target P at $\left(x_{p},r\right)$, the sub-aperture coherent accumulation operation is given by [18]:

(18) $$\begin{array}{cc} S_{u}\left(x_{sub},r\right) & =\int_{K_{xu}-\Delta K_{xa}/2}^{K_{xu}+\Delta K_{xa}/2}S_{f}\left[X^{*}\left(x_{p}\right),x_{p},r\right]\cdot \exp\left\{j\Phi_{m}\left[X^{*}\left(x_{sub}\right),x_{sub},r\right]\right\}dK_{x}\\ & \approx \int_{K_{xu}-\Delta K_{xa}/2}^{K_{xu}+\Delta K_{xa}/2}\exp\left\{jK_{x}\left[X^{*}\left(x_{sub}\right)-X^{*}\left(x_{p}\right)\right]\right\}dK_{x}\\ & \approx \int_{K_{xu}-\Delta K_{xa}/2}^{K_{xu}+\Delta K_{xa}/2}\exp\left[jK_{x}\left(x_{sub}-x_{p}\right)\right]dK_{x} \end{array}$$

where, $S_{u}\left(x_{sub},r\right)$ denotes the coarse imaging result of the *u*th sub-aperture. It is clear in Equation (18) that one needs to calculate the coarse resolution image point-by-point with the sub-aperture back-projection integral. Sub-aperture processing reduces the computational burden at some level. Observing the AMF phase expression in Equation (16), $\Phi_{m}\left(K_{x},x_{p},r\right)$ is a function with respect to the azimuth wavenumber $K_{x}$. In each sub-aperture integral, since the wavenumber spectrum length $\Delta K_{xa}$ is short,$\Phi_{m}\left(K_{x},x_{p},r\right)$ can be approximated into a linear function with respect to $K_{x}$. The approximation expression of Equation (16) is given by:

(19) $$\Phi_{m}\left(K_{x},x_{p},r\right)\approx a_{u}\left(x_{p}\right)\left(K_{x}-K_{xu}\right)+\Phi_{m}\left(K_{xu},x_{p},r\right)$$

where notation *u* still denotes the *u*th sub-aperture and $a_{u}$ denotes the slope of AMF phase of the *u*th sub-aperture. The sub-aperture back-projection integration in Equation (18) is transformed as:

(20) $$S_{u}\left(x_{sub},r\right)=\int_{K_{xu}-\Delta K_{xa}/2}^{K_{xu}+\Delta K_{xa}/2}S_{f}\left(K_{x},x_{p},r\right)\cdot \exp\left[ja_{u}\left(x_{sub},r\right)K_{x}\right]\cdot \exp\left[j\Phi_{m}\left(K_{xu},x_{sub},r\right)\right]dK_{x}$$

One can note that AMF phase slope $a_{u}$ is slowly varying along azimuth coordinate point $x_{sub}$, because of the presence of azimuth-variant motion error. Therefore, $a_{u}$ is able to be linearly approximated with respect to the variable $x_{sub}$, and Equation (20) can be written as follows:

(21) $$S_{u}\left(x_{sub},r\right)=S_{ul}\left(x_{sub},r\right)\cdot \exp\left[j\Phi_{m}\left(K_{xu},x_{sub},r\right)\right]$$

where, $S_{ul}\left(x_{sub},r\right)$ is the sub-aperture back-projection integral formula given by:

(22) $$S_{ul}\left(x_{sub},r\right)=\int_{K_{xu}-\Delta K_{xa}/2}^{K_{xu}+\Delta K_{xa}/2}S_{f}\left(K_{x},x_{p},r\right)\cdot \exp\left[jb_{u}\left(r\right)x_{sub}K_{x}\right]\cdot \exp\left[jb_{u0}\left(r\right)K_{x}\right]dK_{x}$$

where, $b_{u}$ is the monomial coefficient of $a_{u}$ with respect to $x_{sub}$, and $b_{u0}$ is a constant term. It can be found that the integral formula in Equation (22) is regarded as a scaling Fourier transform with scale a factor of $-b_{u}$ and initial phase of $-b_{u0}$, so the integral operation in Equation (22) can be substituted by the chirp-Z transform, which can enhance the computation efficiency in implementation of the sub-aperture back-projection integral. The scale factor can be straightforwardly obtained by twice differential operations on $\Phi_{m}\left(K_{x},x_{sub},r\right)$.

(23) $$b_{u}\left(r\right)=\frac{\partial }{\partial x_{sub}}\left[\frac{\partial }{\partial K_{x}}\Phi_{m}\left(K_{x},x_{sub},r\right)\right]$$

where operational symbol ∂ denotes the differential operator. Then, chirp-Z transform can be introduced in each sub-aperture integral to obtain a set of coarse resolution images. Residual azimuth-variant motion error for each point coordinate is completely compensated for in these sub-aperture back-projection integrals. Substituting Equations (22) into (21), a series of sub-aperture coarse resolution images are obtained.

The next question is how to combine these coarse resolution images into a full resolution image. There is an effective method fusing these sub-images in the azimuth wavenumber domain. As to the *u*th sub-image $S_{u}\left(x_{sub},r\right)$, transforming the sub-aperture images back to the azimuth wavenumber domain.

(24) $$S_{uf}\left(K_{x},r\right)=\int_{-x_{a}/2}^{x_{a}/2}S_{u}\left(x_{sub},r\right)\exp\left(-jK_{x}x_{sub}\right)dx_{sub}$$

Substitute the expression in (18) into (24), the azimuth wavenumber spectrum of sub-image is expressed as:

(25) $$S_{uf}\left(K_{x},r\right)=\mathrm{rect}\left(\frac{K_{x}-K_{xu}}{\Delta K_{xa}}\right)\cdot \exp\left(-jK_{x}x_{p}\right)$$

It is shown in Equation (25) that the center of azimuth wavenumber spectrum is $K_{xu}$. One can obtain the full spectrum by sequentially stitching the sub-aperture azimuth wavenumber spectrum. Therefore, with the full wavenumber spectrum, the fine resolution image is obtained by an inverse Fourier transform to the full azimuth wavenumber spectrum.

(26) $$S\left(X,r\right)=\int_{\Delta K_{x}/2}^{\Delta K_{x}/2}\sum_{u=1}^{U}S_{uf}\left(K_{x},r\right)\cdot \exp\left(jK_{x}\cdot X\right)dK_{x}$$

**Figure 3:**
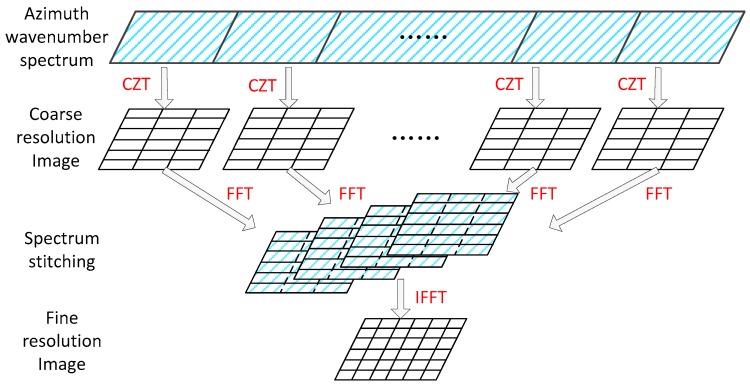
Schematic diagram of frequency domain back-projection algorithm (FDFBPA).

In terms of clarify, the FDFBPA procedure is represented in Figure 3. It is clear that FDFBPA is designed to remove the residual azimuth-variant motion error directly from the azimuth wavenumber sub-aperture back-projection integral strategy, and the integral operations in the coarse resolution imaging process are substituted by a series of CZT operations yielding both enhanced robustness and efficiency.

## 4. Algorithm Description and Analysis

A. Algorithm Flow Description

**Figure 4:**
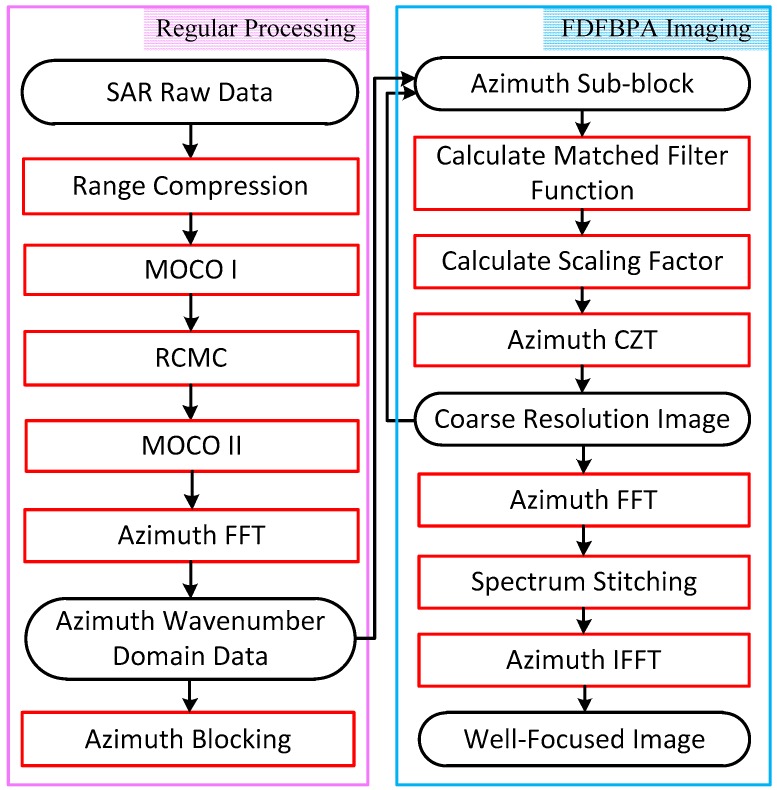
Flowchart of the measured data processing based on FDFBPA.

According to the theoretical analysis in Section 3, we develop a complete SAR imaging flowchart with FDFBPA given in Figure 4. The whole procedure is divided into two main stages, regular processing stage and FDFBPA imaging stage. Some key steps are described as follows:

(a) Range compression. This step is achieved by range-matched filtering. After range compression, one-dimensional imaging is completed.
(b) Coarse MOCO. This step is achieved by two-step MOCO. MOCO I is bulk compensation which compensates the range- and azimuth-invariant motion errors, and MOCO II compensates the residual range-variant motion error. The residual azimuth-variant motion errors would be significant enough to induce distinctive azimuth blurring. According to the principle of two-step MOCO, MOCO I is processed before RCMC and MOCO II after RCMC. Two-step MOCO can also be replaced by some improved MOCO strategies to obtain a better performance in range cell migration correction [26,27].
(c) RCMC. This step is the core of regular processing stage, which is used to correct the range curve in the data. The most commonly applied RCMC schemes include range-Doppler algorithm (RDA), chirp scaling algorithm (CSA) and Omega-k algorithm.
(d) Azimuth blocking in wavenumber domain. After Range compression, RCMC and two-step MOCO, we obtain the azimuth wavenumber domain data by applying an azimuth fast Fourier transform (FFT). The proposed azimuth wavenumber sub-aperture processing strategy can then be introduced. The data need to be partitioned uniformly in the azimuth wavenumber domain, so that we can process the data in azimuth sub-block for the next procedure.
(e) Precise azimuth matched filtering function calculation. In this step, we first build a coarse imaging grid, and calculate a series of precise wavenumber spectrum relative to each point in coarse imaging grid by (13), where the coefficients $a_{0}\cdots a_{4}$ are obtained by fourth order polynomial fitting. Then the precise AMF function is the conjugation of calculated precise wavenumber spectrum and the precise AMF function phase expression as shown in Equation (10).
(f) Scaling factor calculation and azimuth CZT. This step is the core of sub-aperture processing by which we achieve the coarse resolution imaging in this step. Based on the AMF function phase expression, we calculate a scaling factor by Equation (23), then CZT is performed to get a coarse resolution image in each azimuth sub-block.
(g) Spectrum stitching. This step aims at fusing the coarse resolution images into full resolution, which is achieved by azimuth wavenumber spectrum stitching. Specifically, the coarse resolution images are transformed into the wavenumber domain by the azimuth FFT in Equation (24), then a full-resolution and well-focused image is obtained by Equation (26).

B. Computation Analysis

In this section, we consider the calculation burden of the FDFBPA imaging stage, especially the times necessary for the FFT and inverse Fourier transform (IFFT) operations. As shown in Figure 4, with respect to data, the whole azimuth point number is N. We define the coarse resolution imaging grid point number as $N_{a}$, so the compensation step is $N/N_{a}$. For analysis convenience, the azimuth sub-aperture length is also defined as $N_{a}$, so the number of azimuth sub-aperture is $N/N_{a}$. According to the FDFBPA imaging stage of Figure 4, three steps including CTZ, FFT and IFFT operations need to be counted. There are $N/N_{a}$ times $N_{a}$-point CZT, $N/N_{a}$ times $N_{a}$-point FFT and one N-point IFFT, where one $N_{a}$-point CZT operation includes two $N_{a}$-point FFTs and one $N_{a}$-point IFFT. It might be beneficial to account for the computational burden by calculating floating-point operations (FLOPs). One $N_{a}$-point FFT/IFFT operation contains $5N_{a}\log_{2}\left(N_{a}\right)$ FLOPs, so the total FLOP times is given by:

(27) $$\begin{array}{cc} C & =N/N_{a}\cdot \left(2+1\right)\cdot 5N_{a}\log_{2}\left(N_{a}\right)+N/N_{a}\cdot 5N_{a}\log_{2}\left(N_{a}\right)+5N\log_{2}\left(N\right)\\ & =\frac{20N}{N_{a}}\log_{2}\left(N_{a}\right)+5N\log_{2}\left(N\right) \end{array}$$

C. Constraint Condition Analysis

According to the computation analysis above, it is clear that the calculation burden will be reduced with the increase of azimuth sub-aperture length $N_{a}$, but $N_{a}$ cannot constantly increase to pursue high computation efficiency. As illustrated in Section 3, the core of FDFBPA is based on a linearly approximation of AMF phase $\Phi_{m}$ in sub-aperture, so azimuth sub-aperture length $N_{a}$ is constrained by the condition of this approximation. In most cases, the phase error is considered less than π/16 to ensure algorithm stability, so the restriction of $N_{a}$ is given by:

(28) $$\max\left[\begin{array}{l} \left|\Phi_{m}\left(K_{xu}+\frac{N_{a}}{2N}K_{a}\right)-\Phi_{m}\left(K_{xu}\right)-a_{u}\left(\frac{N_{a}}{2N}K_{a}\right)\right|,\\ \left|\Phi_{m}\left(K_{xu}-\frac{N_{a}}{2N}K_{a}\right)-\Phi_{m}\left(K_{xu}\right)-a_{u}\left(\frac{N_{a}}{2N}K_{a}\right)\right| \end{array}\right]\leq \frac{\pi }{16}$$

where $\forall K_{xu}=n\cdot \frac{N_{a}}{N}K_{a}$ and n is an integer with a range of $n\in \left(-\frac{N}{2N_{a}}+1,\cdots ,\frac{N}{2N_{a}}-1\right)$.

## 5. Simulated and Real Data Experiments

A. Experiments with Simulated Data

**Figure 5:**
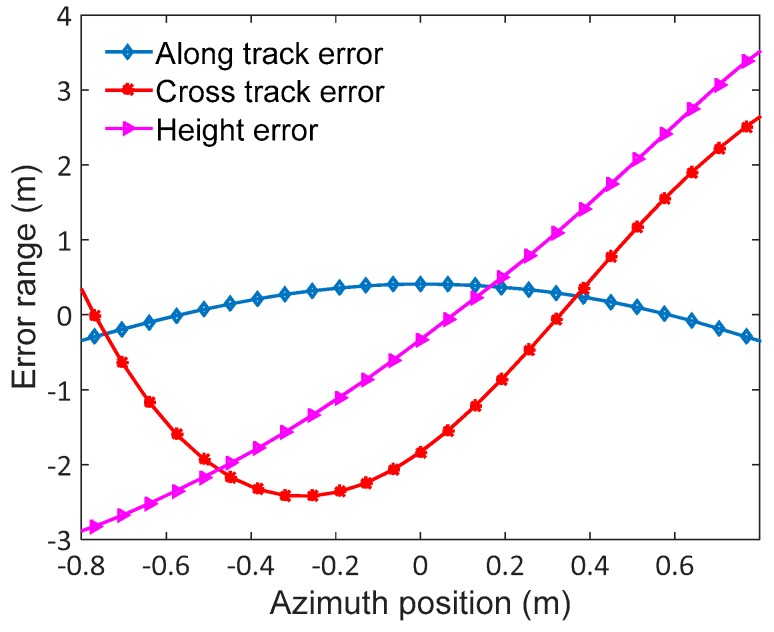
Trajectory deviations for simulation.

**Figure 6:**
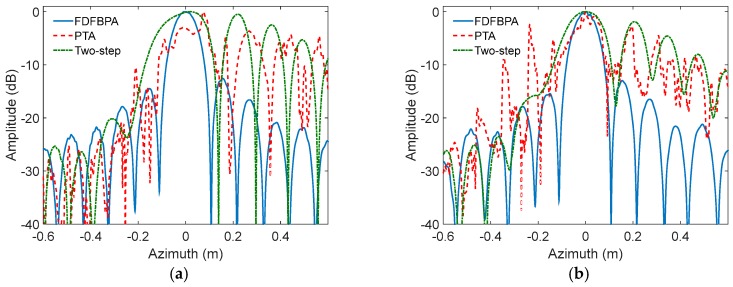
Azimuth impulse response curve comparison of FDFBPA, 25% overlapped PTA and Two-step MOCO: (**a**) 0 degree of squint angle; (**b**) 40 degrees of squint angle.

In order to validate the theory and analysis illustrated in the previous sections, we describe an experiment performed with simulated Ka-band SAR data in this subsection. The main SAR system parameters are shown in Table 1. In this experiment, two points are simulated in different squint angles with trajectory deviations, which will cause azimuth-variant motion error significant enough to blur the azimuth impulse response curve. The trajectory deviations are extracted from real-measured INS data shown in Figure 5. The data contain 8192 pulses in azimuth direction. For the propose of comparing the compensation performance, FDFBPA, PTA and two-step MOCO approaches are implemented to focus the points. Due to the fact that the azimuth-variance of motion error in azimuth direction is serious, the sub-aperture length is set as 16 points with 25% overlap. The azimuth impulse response curve comparisons of FDFBPA, PTA and two-step MOCO are shown in Figure 6, where Figure 6a is at 0 degrees of squint angle, and Figure 6b is at 40 degrees. It is shown that azimuth-variant MOCO may cause serious defocusing in azimuth direction, so two-step MOCO is blurred in the figures. PTA also fails to refocus the points because the shortcomings of the post-filtering strategy; data blocking in the image domain seriously disrupted the focusing performance. FDFBPA is able to refocus the points well for comparison. In order to quantitatively evaluate the focused improvement of the proposed algorithm compared with the other algorithms, three quantitative metrics are introduced to measure the point impulse responses, which are peak sidelobe ratio (PSLR), integrated sidelobe ratio (ISLR) and impulse response width (IRW). The statistical results are shown in Table 2. We demonstrate that the PSLR and ISLR of PTA are both large, because necessary extension is absent in the block length and overlapping part. Furthermore, the inevitable split of energy in the image domain would cause emergence of multiple peaks. In contrast, FDFBPA applies a blocking strategy in the frequency domain so it performs in a robust manner in the face of strong motion errors.

B. Experiments with Real-Measured Data

**Figure 7:**
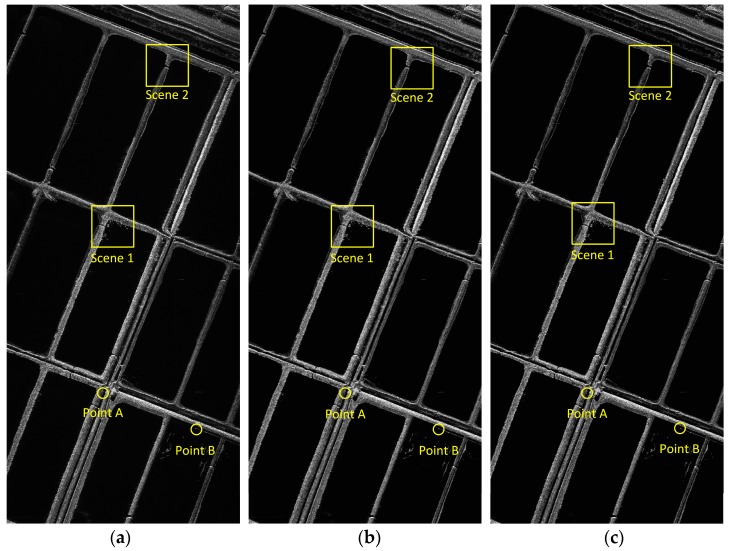
Imaging result processed with different algorithms: (**a**) FDFBPA; (**b**) PTA; (**c**) Two-step MOCO.

**Figure 8:**
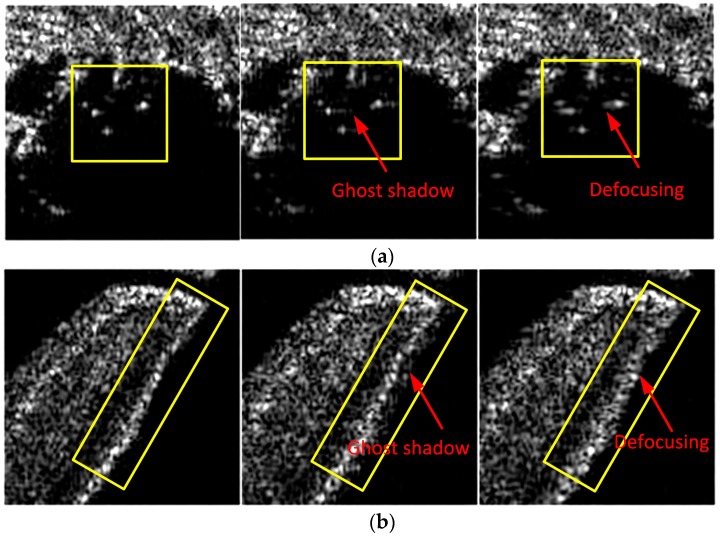
Comparison of three algorithms in Scene 1 and Scene 2: (**a**) Comparison of Scene 1 (left to right: FDFBPA, PTA and Two-step MOCO); (**b**) Comparison of Scene 2 (left to right: FDFBPA, PTA and Two-step MOCO).

In this subsection, two sets of comparison experiments are provided based on the processing of measured data recorded by an experimental airborne Ka-band SAR system. The first experiment aims at broad side operating modes. Some main SAR system parameters are shown in Table 1, where the squint angle is less than two degrees with a resolution of 0.15 m in both range and azimuth. The instantaneous position and motion parameters of the platform are measured by a high-accuracy INS equipped on the platform, the azimuth-variant motion error during the data collection is severe enough to cause defocusing of the image. A set of imaging results processed by the FDFBPA, 25% overlapped PTA and two-step MOCO are shown in Figure 7. In these images, two typical areas with obvious artificial structures are marked by yellow rectangles, which are amplified in Figure 8a,b for comparison. The sub-block length for processing is 16 points. It is clear that there are ghost shadows appearing in PTA results for the reason that the sub-aperture length is too short to meet the refocus conditions in Equations (14) and (15). Furthermore, the defocusing of two-step MOCO results are also significant because the residual azimuth-variant motion error remains.

**Figure 9:**
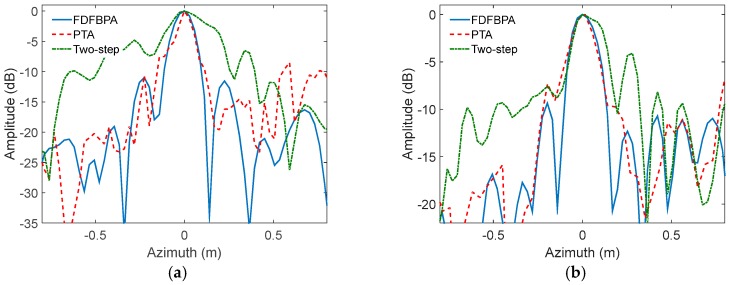
Azimuth pulse response curve comparison of FDFBPA, PTA and Two-step MOCO. (**a**) Scatter Point A; (**b**) Scatter Point B.

**Figure 10:**
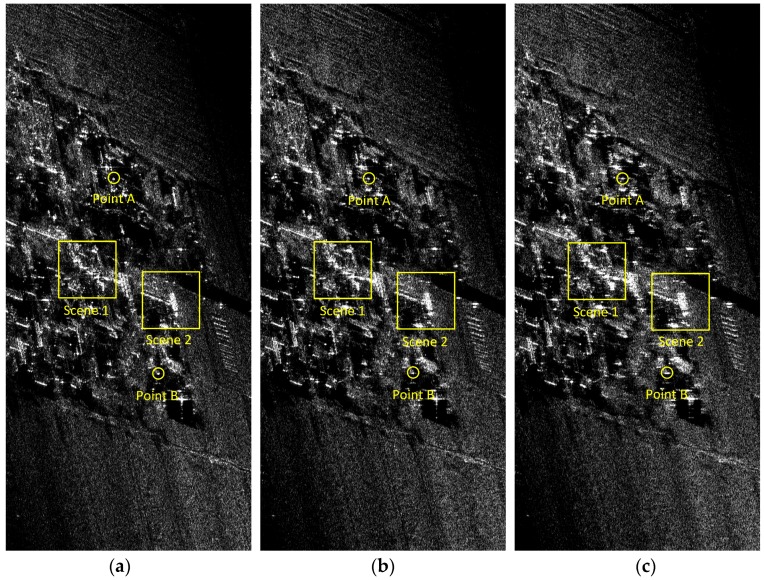
Imaging result processed with different algorithms (**a**) FDFBPA; (**b**) PTA (**c**) Two-step MOCO.

In order to check the azimuth point impulse response improvement of the FDFBPA algorithm, two isolated point-like targets named point A and point B are extracted from Figure 7 by yellow circles for azimuth point impulse response function comparison, and are shown in Figure 9a,b respectively. Sidelobes of PTA and two-step MOCO are obviously higher than FDFBPA, the presence of high sidelobes causes image ghosting and raises the floor noise of the SAR image. The quantitative analysis results of the azimuth point spreading response functions of Figure 10 are listed in Table 3. It is evident that serious distortion and smearing occur in both PTA and two-step MOCO results, while only the FDFBPA results provide a well-focused performance. From the comparison of the local scene images and point target impulse responses, one can conclude that FDFBPA achieves significant improvements in focusing data with strong motion errors. With the point-to-point correction precision of azimuth-variant motion error phase terms, FDFBPA performs a more robust manner than PTA with the image domain post-filtering strategy.

**Figure 11:**
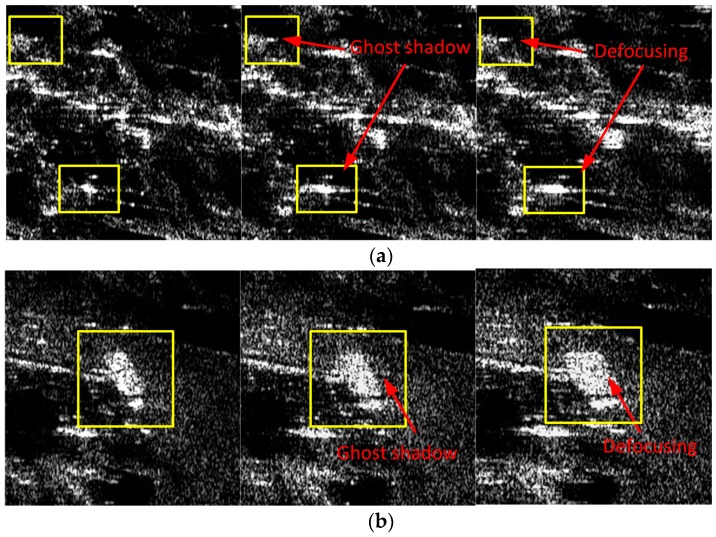
Comparison of four algorithms in Scene 1 and Scene 2: (**a**) Comparison of Scene 1 (left to right: FDFBPA, PTA and Two-step MOCO); (**b**) Comparison of Scene 2 (left to right: FDFBPA, PTA and Two-step MOCO).

**Figure 12:**
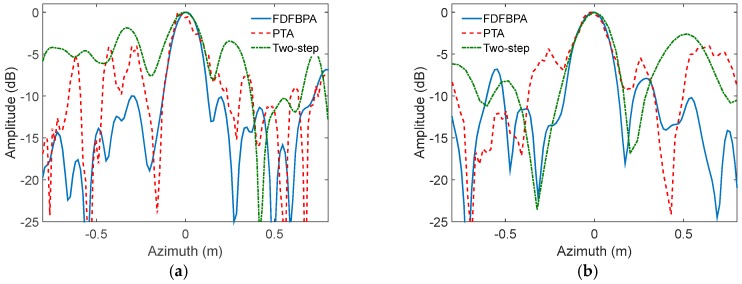
Azimuth pulse response curve comparison of FDFBPA, PTA and Two-step MOCO: (**a**) Scatter Point A; (**b**) Scatter Point B.

In the second experimental set, the radar is working at a high squint angle of about 40 degrees with a resolution of 0.15 m in both range and azimuth; some main system parameters are shown in Table 1. The range and azimuth coupling is firstly removed by range walk correction (RWC) processing, and we can regard the processed data as obtained in broad side mode. The precise azimuth wavenumber spectrum needs to be recalculated considering the influence of RWC, which has been previously worked out in [28], and will not be discussed here. According to the precise azimuth wavenumber spectrum, a set of imaging results processed by the FDFBPA, 25% overlapped PTA and two-step MOCO are shown in Figure 10, with sub-block length of 16 points. Two typical areas marked by yellow rectangles are amplified for comparison and shown in Figure 11a,b. Furthermore, two isolated point-like targets named points A and point B are extracted from Figure 10 by yellow circles for azimuth point impulse response function comparison, which are shown in Figure 12a,b respectively. Statistical indicators like PSLR, ISLR and IRW are used to numerically measure the impulse response function performance (Table 4). Two-step MOCO can effectively remove the range-variant motion errors, but cannot correct the azimuth-variant phase terms, which is more significant in highly squinted imaging modes. Therefore, the two-step result is severely blurred with error phases. PTA seems to partly compensate for the defocusing by image domain post-filtering, however, the sub-block length and overlapped part are not long enough to meet the refocusing condition. As a result, the PTA results suffer from ghost shadows that would decrease imaging performance. FDFBPA performs the SAR imaging process by a point-to-point strategy in the sub-aperture focusing stage, which provides overwhelming precision and robust improvements in correcting strong motion errors.

For the propose of testing the calculation improvement of FDFBPA compared with PTA, we record the calculation time for the two algorithms under different sliding step factors. The sliding step here means the interval between centers of adjacent sub-blocks for PTA, and also means the interval of coarse resolution grid. According to the analysis in Section 3, we know that with the lengthening of sliding step, block number of FDFBPA is increasing while the block number of PTA is decreasing correspondingly. In order to get the experiment closer to the actual situation, sub-block length of PTA is 64, and the overlapped part depending on the sliding step range block length is 16 points without overlap. The computer platform is installed with Windows 7 64-bitoperating system, E5-2643@3.3GHz CPU, 32-GBmemory and Matlab with version of R2015a. A block of 3072 × 16384 (range × azimuth) points SAR data is used for test. The computation time comparison results are shown in Table 5. It is shown that, with the help of CZT operation and without sub-aperture overlapping, FDFBPA processes a much higher operation efficiency compared with PTA under short sliding steps. It is worth to explaining that the computation time of PTA decreases with the increase of sliding steps for the reduction of sub-block number. In contrast, the computation time of FDFBPA is slowly growing with the increase of sliding step due to the fact that we approximately use CZT for sub-aperture fast imaging, and the computation time mainly depends on the sub-aperture block number. However, we cannot infinitely shorten the interval of coarse resolution grid for FDFBPA to pursue a higher computational speed, because the sub-aperture length would be correspondingly extended so that the phase linear approximation condition in Equation (28) would be destructed.

## 6. Conclusions

Focusing on the precise azimuth-variant MOCO for airborne SAR, a frequency domain fast back-projection algorithm named FDFBPA is proposed in this paper to deal with strong azimuth-variant motion errors. Based on the analysis of image domain post-filtering strategy such as PTA, it is known that PTA has to make a balance between reduction in imaging quality and increased calculations. FDFBPA is designed with both high precision and high efficiency for imaging with strong motion errors. FDFBPA disposes of the azimuth-variant motion errors by precise azimuth wavenumber spectrum calculation. Moreover, with the utilization of the wavenumber domain sub-aperture processing strategy and CZT operation, the efficiency of the algorithm is further improved. Simulated and real-measured data experiments show that the proposed FDFBPA is more robust for imaging with strong motion errors compared with PTA, and the efficiency of FDFBPA for processing real measured data is also verified.

## Figures and Tables

**Table 1:** SAR System Parameters.

Parameter	Value
Carrier frequency	35 GHz
Bandwidth	900 MHz
Center slant range	5000 m
Coherent Processing Interval	1.5 s
Ideal velocity	70 m/s
Pulse repetition frequency	5000 Hz

**Table 2:** Comparison of Quantification Statistics Results.

Approach	0 Degree of Squint Angle			40 Degrees of Squint Angle		
	PSLR (dB)	ISLR (dB)	IRW (m)	PSLR (dB)	ISLR (dB)	IRW (m)
FDFBPA	−12.4113	−10.5214	0.1313	−12.4936	−10.5719	0.1312
PTA	−4.4790	−0.8512	0.1451	−2.3360	−2.7938	0.1489
Two−step	−0.4803	−0.9381	0.2199	−1.8899	−3.3940	0.2000

**Table 3:** Comparison of Quantification Statistics Results.

Approach	Point A			Point B		
	PSLR (dB)	ISLR (dB)	IRW (m)	PSLR (dB)	ISLR (dB)	IRW (m)
FDFBPA	−10.9869	−8.5942	0.1968	−9.3550	−6.1651	0.2531
PTA	−8.5014	−5.6829	0.1968	−5.1406	−2.9050	0.2531
Two−step	−4.8080	−4.3006	0.3937	−4.1109	−2.8496	0.2812

**Table 4:** Comparison of Quantification Statistics Results.

Approach	Point A			Point B		
	PSLR (dB)	ISLR (dB)	IRW (m)	PSLR (dB)	ISLR (dB)	IRW (m)
FDFBPA	−9.9872	−7.4124	0.2152	−7.8962	−7.1813	0.2583
PTA	−0.5440	3.9724	0.2439	−4.0268	−0.9475	0.2583
Two−step	−3.0850	−2.3533	0.2870	−2.7590	−3.9529	0.3214

**Table 5:** Computation time comparison of FDFBPA and PTA under different sliding step.

Approach	Sliding Step (Points)		
	8	16	32
FDFBPA	159.58 s	192.75 s	243.67 s
PTA	1427.24 s	725.41 s	423.05 s
